# Spindle cell squamous cell carcinoma exhibiting prominent neutrophil phagocytosis: a case report

**DOI:** 10.1186/s13256-021-03066-z

**Published:** 2021-08-27

**Authors:** Manabu Yamazaki, Satoshi Maruyama, Tatsuya Abé, Yoshimasa Sumita, Yuji Katsumi, Yutaka Nikkuni, Takafumi Hayashi, Jun-ichi Tanuma

**Affiliations:** 1 Division of Oral Pathology, Faculty of Dentistry and Niigata University Graduate School of Medical and Dental Sciences, 2-5274 Gakkocho-dori, Chuo-ku, Niigata, 951-8514 Japan; 2 Oral Pathology Section, Department of Surgical Pathology, Niigata University Hospital, Niigata, Japan; 3 Division of Oral and Maxillofacial Surgery, Faculty of Dentistry and Niigata University Graduate School of Medical and Dental Sciences, Niigata, Japan; 4 Division of Oral Radiology, Faculty of Dentistry and Niigata University Graduate School of Medical and Dental Sciences, Niigata, Japan

**Keywords:** Spindle cell squamous cell carcinoma, Oral cavity, Neutrophil, Phagocytosis, Cell-in-cell

## Abstract

**Background:**

Spindle cell squamous cell carcinoma is an uncommon variant of squamous cell carcinoma; its diagnosis is sometimes challenging because it histopathologically resembles neoplastic or reactive spindle cell lesions of mesenchymal origins. Here, we report a rare case of spindle cell squamous cell carcinoma exhibiting prominent neutrophil phagocytosis.

**Case presentation:**

A 69-year-old Japanese man presented with pain and a polypoid mass on the lower left gingiva. He had received chemoradiotherapy for squamous cell carcinoma of the buccal mucosa 15 years prior to this consultation. In addition, he was treated for mandibular osteonecrosis 6 years after chemoradiotherapy without evidence of cancer recurrence. A biopsy revealed atypical spindle or pleomorphic cells scattered in the edematous and fibrin-rich stroma; however, no malignant squamous components were apparent. These atypical cells frequently contained neutrophils within their cytoplasm that formed cell-in-cell figures. Immunohistochemically, the atypical cells were negative for cytokeratins, epithelial membrane antigen, and E-cadherin, but positive for p63, vimentin, and p53. Although these findings suggested spindle cell squamous cell carcinoma, it was difficult to reach a definitive diagnosis. Based on a clinical diagnosis of a malignant tumor, the patient underwent a hemimandibulectomy. The surgically resected specimen had a typical spindle cell squamous cell carcinoma histology consisting of biphasic spindle cells and conventional squamous cell carcinoma components. Moreover, the surgical specimen also exhibited spindle tumor cells that frequently included neutrophils, around which intense staining for lysosomal-associated membrane protein 1 and cathepsin B was observed. This suggested that the cell-in-cell figures represent active neutrophil phagocytosis by tumor cells, and not emperipolesis.

**Conclusion:**

The presence of neutrophil phagocytosis may be a potent indicator of malignancy.

## Introduction

Spindle cell squamous cell carcinoma (SCSCC) is an uncommon variant of squamous cell carcinoma (SCC) that is characterized by spindle and/or pleomorphic cells [1]. In the head and neck region, SCSCC commonly occurs in the larynx [2] and oral cavity [3, 4]. The histopathological diagnosis of SCSCC can sometimes be challenging for surgical pathologists when squamous components such as conventional SCC and/or dysplastic epithelia are absent from biopsy specimens, particularly those that are small. In such cases, spindle cell neoplasms such as certain types of sarcoma and reactive lesions should be considered in differential diagnosis.

The cell-in-cell phenomenon observed in malignant tumors is well known to pathologists [5] and is considered a hallmark of malignancy in diagnostic cytology [6]. To date, neutrophils [7], lymphocytes [8], erythrocytes [9], and living [5] or neighboring dead tumor cells [10] have been observed as intracellular tumor cell components. These cell-in-cell figures can be formed by several distinct cellular processes such as phagocytosis (also referred to as cannibalism), emperipolesis (the movement of living cells through the cytoplasm of host cells), and entosis (one cell invading another) [11]. Furthermore, recently, a relationship between the cell-in-cell phenomenon and malignancy has been shown; for example, cell-in-cell formation by entosis causes aneuploidy in human breast tumors [11]. We previously demonstrated that the phagocytosis of apoptotic oral SCC cells by neighboring SCC cells is mediated by activation of Ras-related C3 botulinum toxin substrate 1 (Rac1) and promotes cell migration [12].

Here, we present a patient with a rare SCSCC, characterized by prominent phagocytosis of neutrophils. The tumor originated from the lower gingiva of the patient, who had received chemoradiotherapy for SCC of the buccal mucosa 15 years prior to this consultation. Although we could not reach a definitive diagnosis of SCSCC based on the biopsy specimen, the lesion was finally diagnosed as SCSCC after confirming the concurrent presence of conventional SCC in the hemimandibulectomy specimen.

## Case presentation

**Fig. 1:**
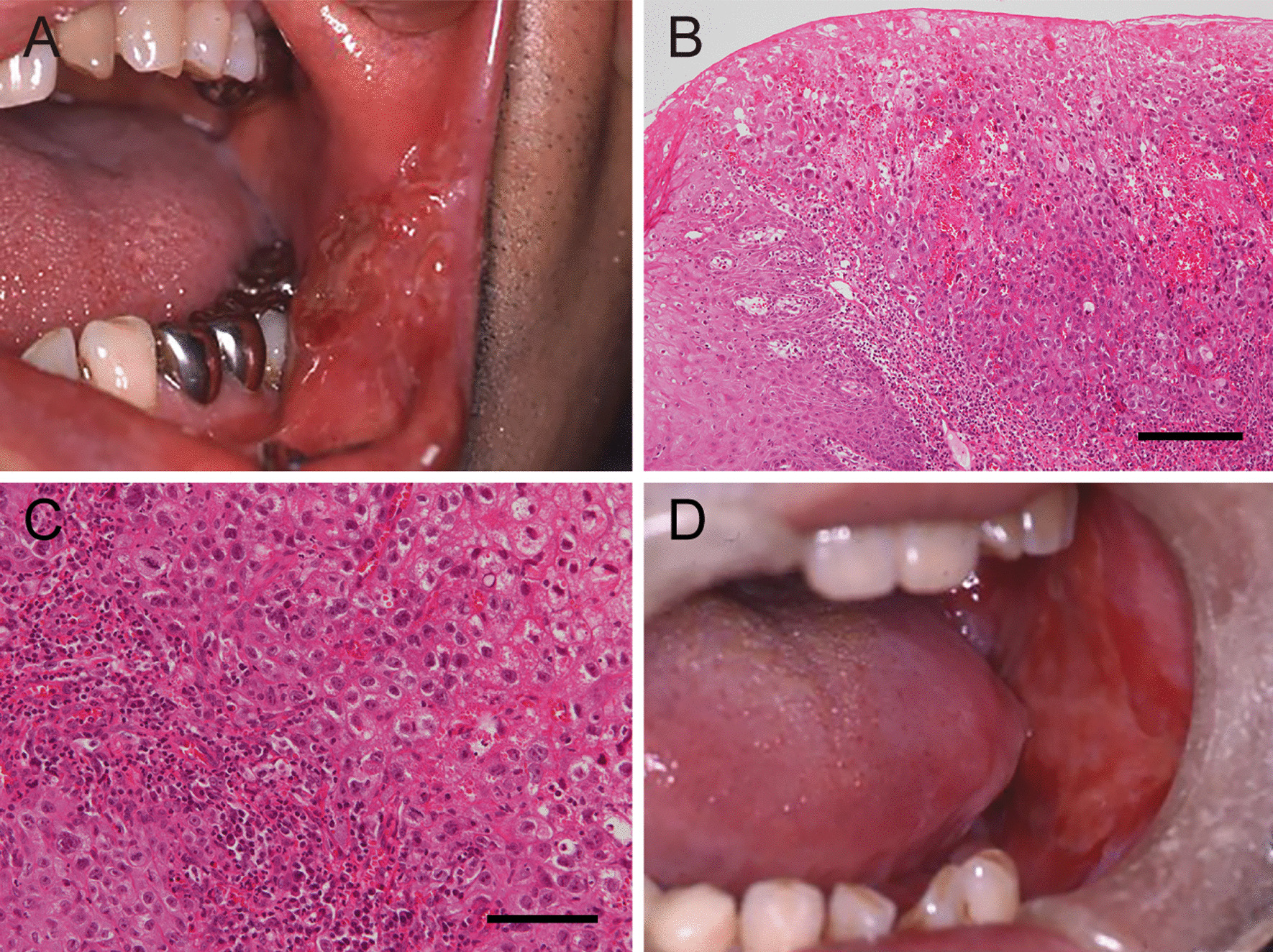
Clinical and histopathological findings of the initial lesion that arose in the left buccal mucosa. **A** Intraoral view at the first examination: the patient presented with a tumor in the left buccal mucosa that had a granular surface and measured 22 × 19 mm. **B**, **C** Histopathological examination of the biopsy specimen revealed squamous cell carcinoma without obvious keratinization (hematoxylin and eosin staining; scale bar 200 μm [**B**] and 100 μm [**C**]). **D** An intraoral view after chemoradiotherapy showed that the tumor had disappeared

A 69-year-old Japanese man presented with pain and a polypoid mass on the lower left gingiva for which he consulted in our hospital. He had received chemoradiotherapy for SCC of the buccal mucosa 15 years prior to this consultation at the age of 54 years. At that time, he had an ulcerative lesion with a reddish and rough surface in the buccal mucosa including the corner of the mouth (Fig. 1A). The patient was a plasterer, and asbestos exposure was not apparent. He had no relevant medical history, including no history of smoking and alcohol consumption. The lesion was diagnosed as moderately differentiated SCC following a biopsy (Fig. 1B, C). Radiological examinations suggested a cancer metastasis in the left submandibular lymph node, and he received chemotherapy with cisplatin (total 110 mg) and 5-fluorouracil (total 2925 mg) via peripheral venous route and concomitant radiotherapy: the primary tumor and neck were irradiated at the dose of 70 Gy, performed in the hospital for 4 months. The treatment resulted in a complete clinical remission of the primary tumor (Fig. 1D) and a complete remission of the neck lesion, which was pathologically confirmed as metastasis composed of nonviable SCC cells (data not shown). No recurrence was detected in subsequent years; however, he developed mandibular osteonecrosis 6 years after chemoradiotherapy and was treated with oral roxithromycin.

**Fig. 2:**
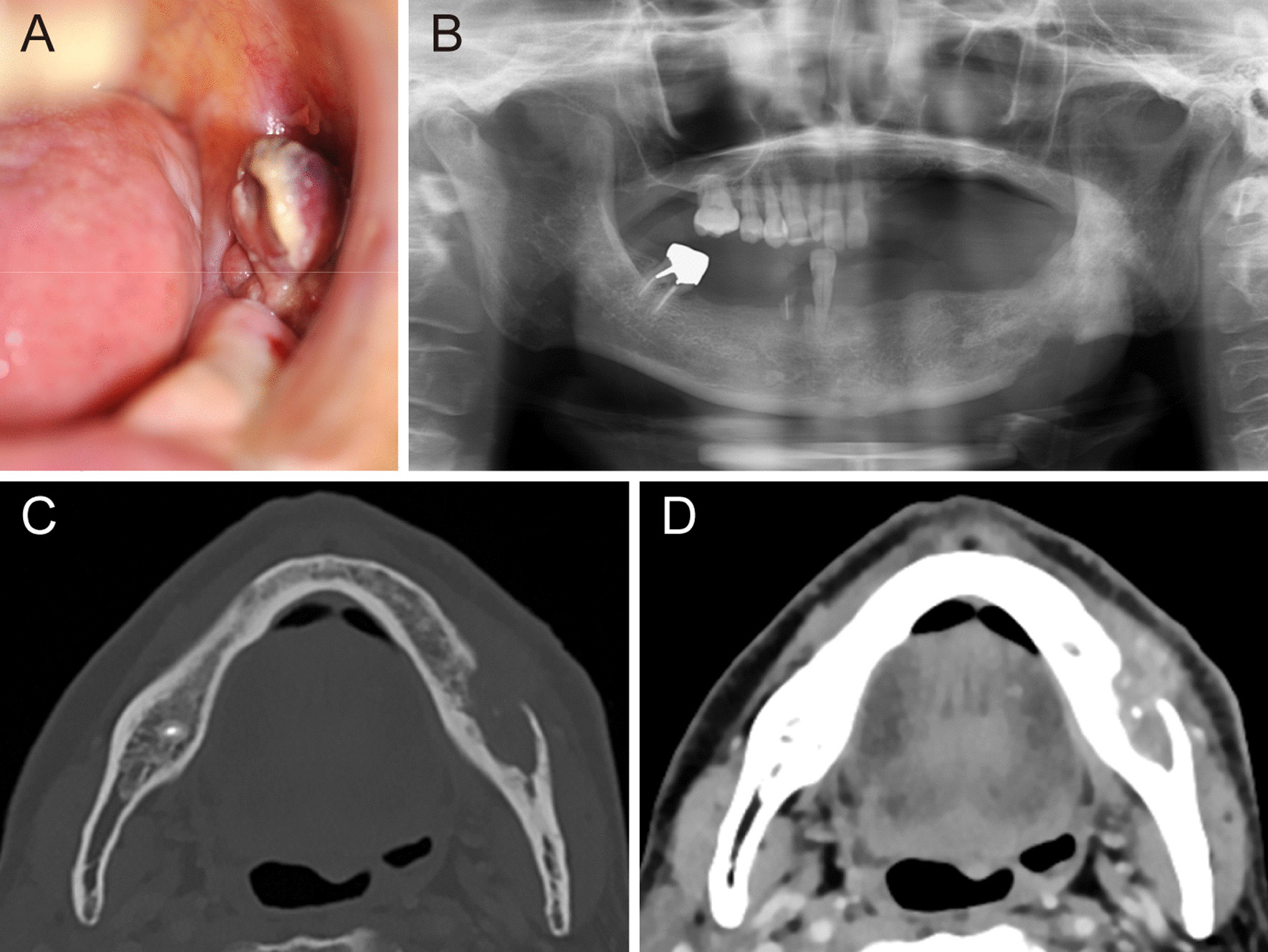
Clinical and radiological findings of the polypoid mass arising in the left molar region of the mandible 15 years later. **A** Intraoral view showing a polypoid mass (10 mm in diameter) with an ulcerated surface in the left lower molar region of the lower gingiva. **B** Panoramic radiography showed a moth-eaten bone resorption pattern. **C** Computed tomography (CT) image, bone window, axial section, and **D** contrast-enhanced CT image, soft-tissue window, axial section revealed an enhancing mass lesion with irregular resorption of the mandible

On examination at the age of 69 years, his vital signs were within normal ranges. He developed Parkinson’s disease a year before this consultation, and the disease was well controlled with oral levodopa (300 mg/day) and benserazide hydrochloride (85.5 mg/day). He showed trismus; however, he had no other abnormal physical and neurological findings. On intraoral inspection, a polypoid mass measuring 10 mm in diameter was found in the left molar region of the lower gingiva (Fig. 2A). Its surface was rough and covered by a whitish pseudomembrane. Panoramic radiography showed bone resorption, exhibiting a moth-eaten appearance that involved the base of the mandible (Fig. 2B). Contrast-enhanced computed tomography revealed a soft-tissue mass lesion accompanied by extensive bone resorption of the mandible (Fig. 2C, D).

**Fig. 3:**
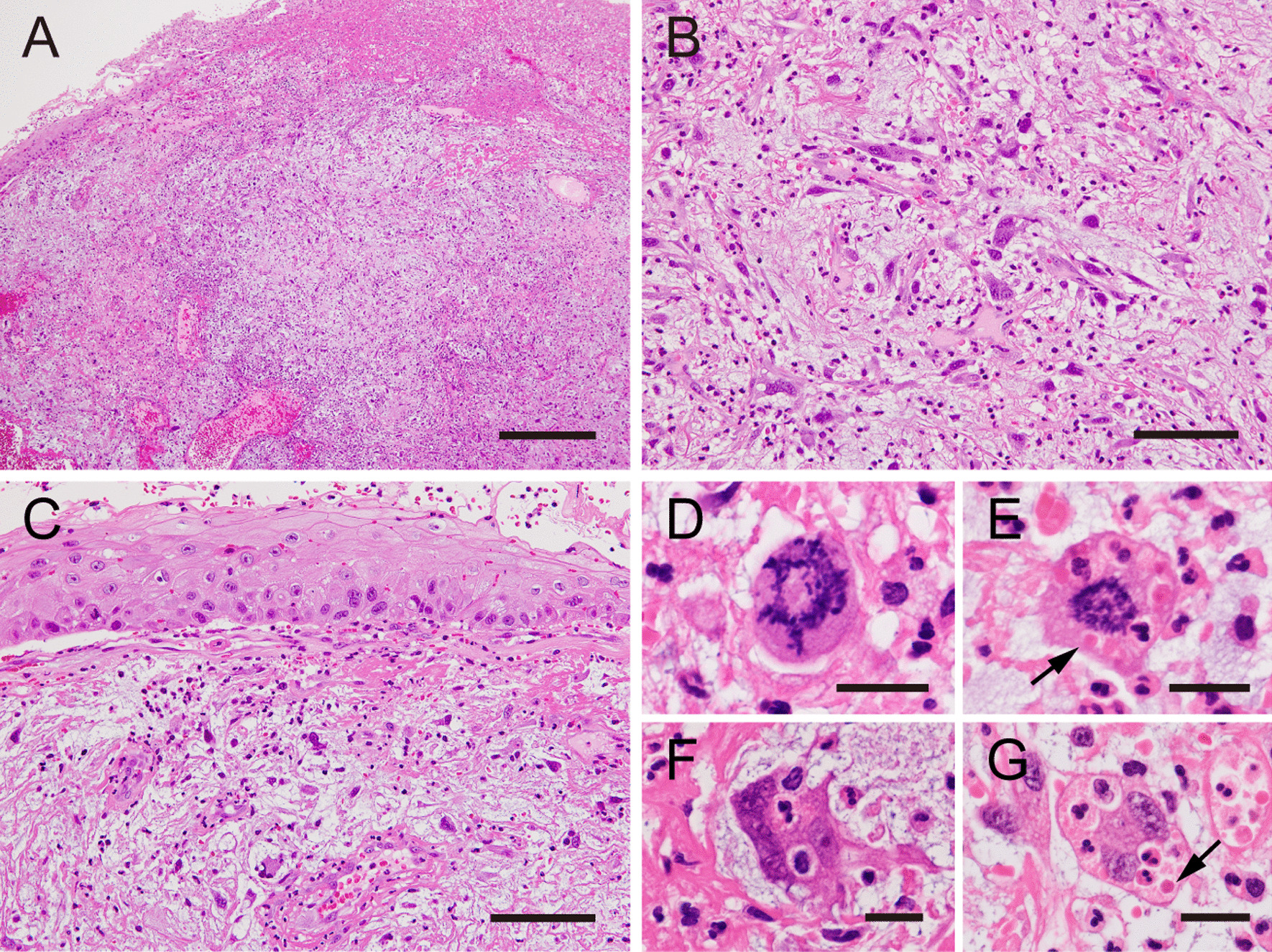
Histopathological findings of the biopsy specimen. **A** The polypoid mass was composed of loose granulation tissue with an ulcerated surface. **B** Atypical spindle cells with pleomorphic nuclei were scattered together with inflammatory cells within the edematous and fibrin-rich stroma. **C** No obvious neoplastic changes were observed in the covering squamous epithelium. **D** A portion of the atypical spindle cells showing atypical mitotic figures. **E**–**G** Neutrophil phagocytosis was frequently observed; some neutrophils lost their nuclei and appeared to be degraded (arrows). **A**–**G** hematoxylin and eosin staining; scale bars: 500 μm (**A**), 100 μm (**B**, **C**), and 20 μm (**D**–**G**)

A malignant tumor was suspected, and a biopsy specimen from the polypoid lesion was obtained. The polypoid mass was composed mainly of loose granulation tissue (Fig. 3A), where atypical spindle or pleomorphic cells were scattered in the fibrin-rich edematous or pale stroma together with neutrophils, lymphocytes, and capillary endothelial cells (Fig. 3B). The surface of the lesion was mostly ulcerated but partially covered by squamous epithelium with no obvious atypia; only slight nuclear enlargement and disordered basal cell polarity were noted (Fig. 3C). In the subepithelial tissue, atypical spindle cells were arranged haphazardly and exhibited large, basophilic cytoplasm and bizarre nuclei (Fig. 3B, C). Some of them exhibited mitotic figures, some of which were atypical (Fig. 3D, E). Neutrophils were frequently incorporated within their cytoplasm (Fig. 3E–G), some of which appeared to be degraded with missing nuclei (Fig. 3E, G, arrows).

**Fig. 4:**
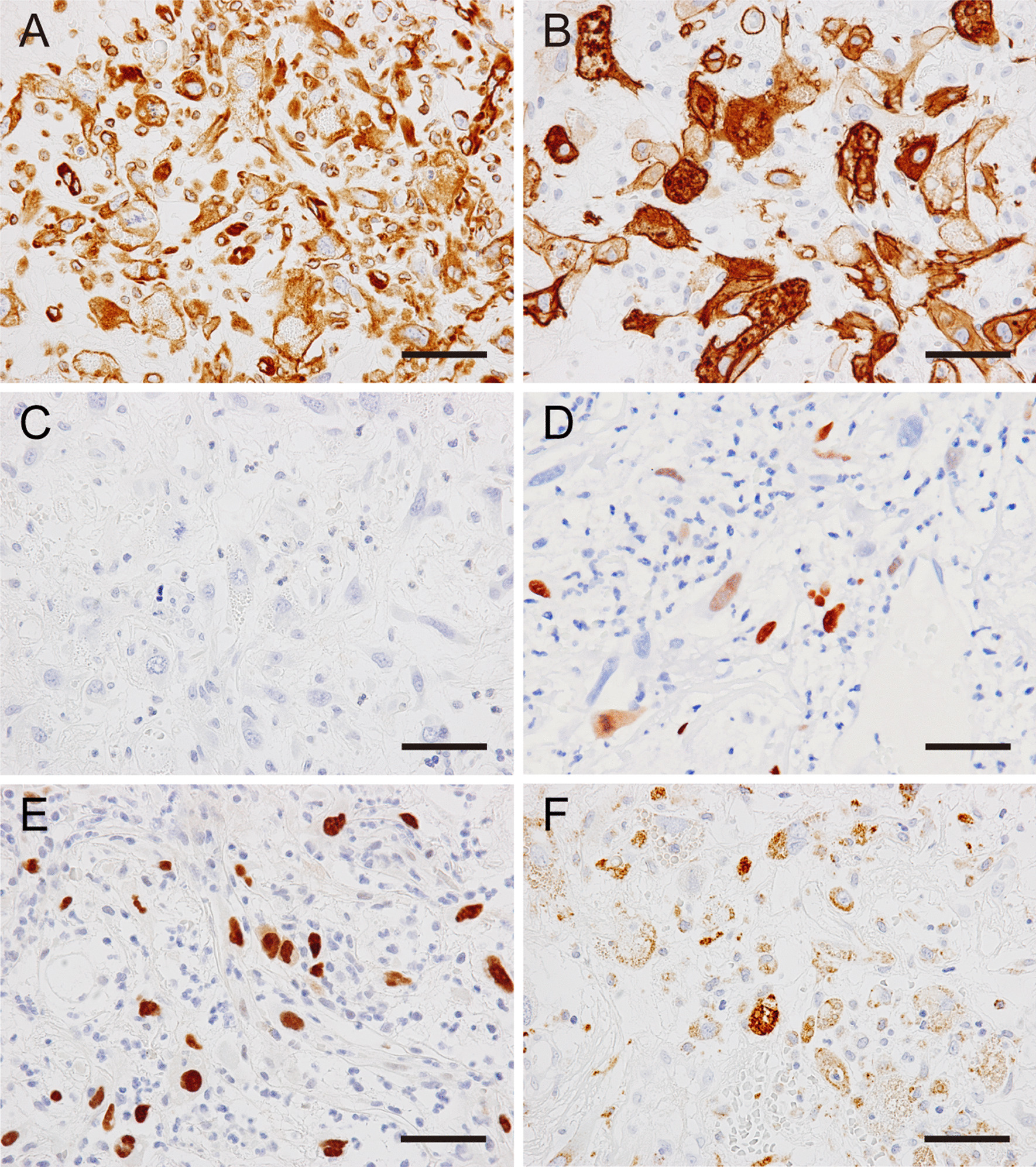
Immunohistochemical analysis of the biopsy specimen using immunoperoxidase staining for vimentin (**A**), α-smooth muscle actin (**B**), pan-cytokeratin (AE1/AE3) (**C**), p63 (**D**), p53 (**E**), and CD68 (**F**); hematoxylin was used as the counterstain. Scale bars: 50 μm (**A**–**F**). Atypical cells were strongly positive for vimentin (**A**) and α-smooth muscle actin (**B**), but not for pan-cytokeratin (**C**). Nuclear staining of p63 (**D**) and p53 (**E**) was observed in a portion of the atypical cells, which also had CD68-positive granules within the cytoplasm (**F**)

Immunohistochemistry revealed that atypical spindle cells were positive for vimentin (Fig. 4A) and α-smooth muscle actin (Fig. 4B), but not for pan-cytokeratin (Fig. 4C). No epithelial markers other than p63 were detected (Table 1). The spindle cells were partially positive for p63 (Fig. 4D), and most were positive for p53 (Fig. 4E). Ki-67 staining was observed in approximately half of the cells (data not shown). Furthermore, granular CD68 staining was observed in the cytoplasm (Fig. 4F). These findings were highly suggestive of a malignancy, and SCSCC and post-irradiation sarcoma were considered in the differential diagnosis, given that the patient had a history of chemoradiotherapy for treating SCC of the buccal mucosa 15 years before.

**Table 1:** Antibodies used in this study and the results of staining

Antigen	Type	Clone	Source	Dilution	Retrieval	Results
pan-CK	M	AE1/AE3	Dako	1:100	CB	−
CK 5/6	M	D5/16 B4	Dako	1:100	CB	−
CK 17	M	E3	Dako	1:100	CB	−
EMA	M	E29	Nichirei Bioscience	Prediluted	None	−
E-cadherin	Rm	24E10	Cell Signaling Technology	1:200	CB	−
p63	M	4A4	Dako	1:50	CB	+
Vimentin	M	Vim 3B4	Dako	1:500	Trypsin	+
α-SMA	M	1A4	Dako	1:100	CB	+
Desmin	M	D33	Dako	1:100	CB	−
S-100	Rp	−	Dako	1:1000	Trypsin	−
CD31	M	JC70A	Dako	1:50	Trypsin	−
CD34	M	NU-4A1	Nichirei Bioscience	1:100	CB	−
CD68	M	PG-M1	Dako	1:50	Trypsin	+
p53	M	DO-7	Dako	1:50	CB	+
Ki-67	M	MIB1	Dako	1:50	CB	+ (approximately 50%)
LAMP-1	M	H5G11	Santa Cruz Biotechnology	1:50	CB	+
Cathepsin B	Rm	D1C7Y	Cell Signaling Technology	1:500	CB	+
Neutrophil elastase	M	NP57	Dako	1:50	None	−

*CK* cytokeratin, *EMA* epithelial membrane antigen, *SMA* smooth muscle actin, *M* mouse monoclonal, *Rp* rabbit polyclonal, *Rm* rabbit monoclonal, *CB* autoclave treatment with citrate buffer (pH 6), *LAMP-1* lysosomal-associated membrane protein 1.

Although the final diagnosis had not been determined, the patient was admitted to our hospital for surgery. On admission, his height and body weight were 145 cm and 45.7 kg, respectively, and vital signs were within normal range (blood pressure 127/79 mmHg, pulse 68 beats/minute, and body temperature 36.0 °C). Complete blood count showed mild anemia (red blood cells 4.07 × 10^12^/L, hemoglobin 128 g/L, hematocrit 36.0%, mean corpuscular volume 90.4 fL, mean corpuscular hemoglobin 31.4 pg, mean corpuscular hemoglobin concentration 348 g/L, white blood cells 5.49 × 10^9^/L, and platelets 130 × 10^9^/L). The biochemical test results of blood and urine were within normal ranges with the exception of a mild elevation in C-reactive protein (8.7 mg/L), which was presumably caused by the gingival tumor. He underwent hemimandibulectomy pursuant to a clinical diagnosis of a malignant tumor. The postoperative course was uneventful.

**Fig. 5:**
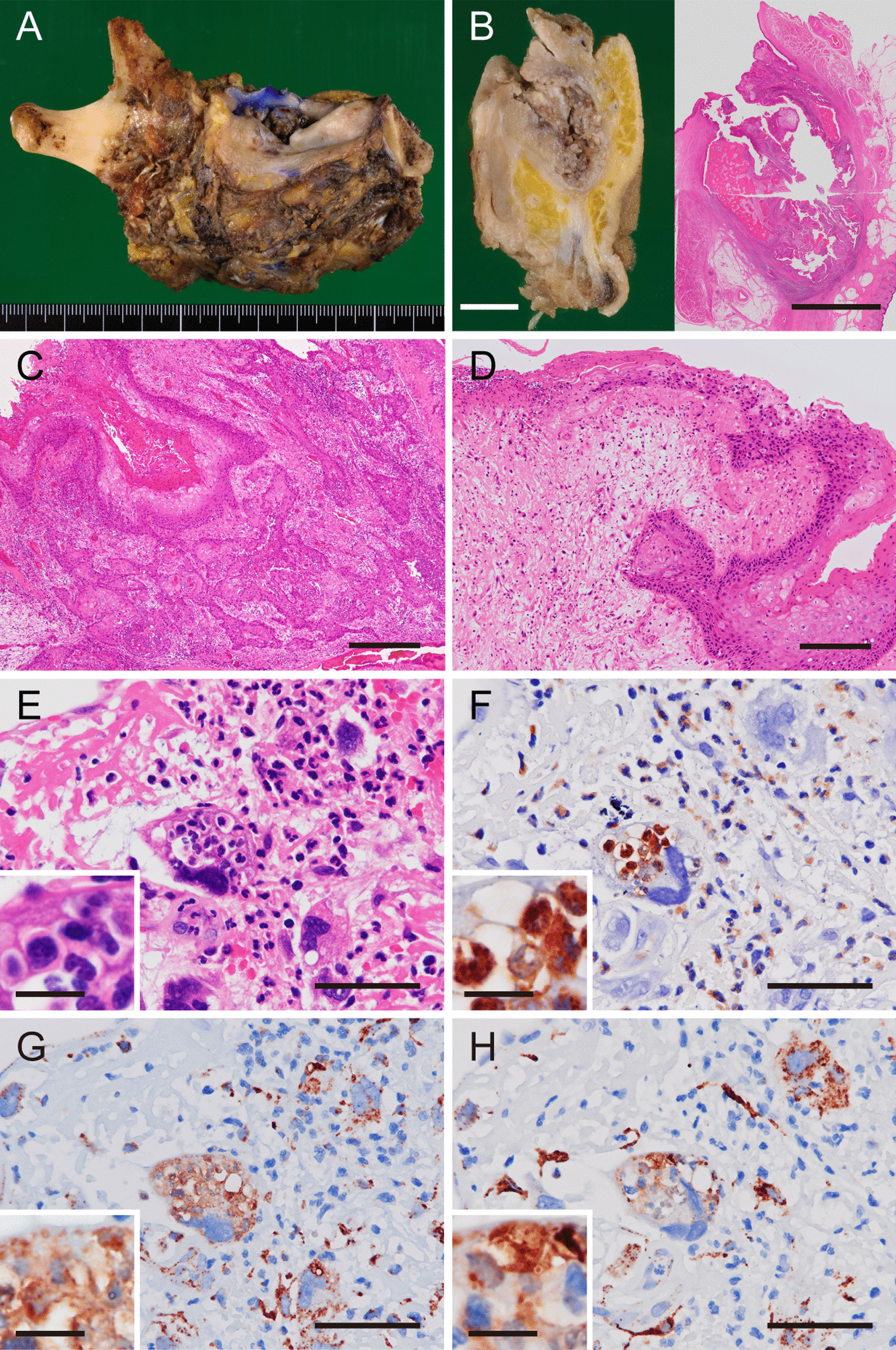
Pathological findings in the surgically resected specimen. On gross viewing, a protruded lesion with an ulcerated surface was observed in the gingiva of the left molar region (**A**). On the cut surface of the specimen (**B**, left panel), a whitish solid tumor invading the mandibular bone was evident; the right panel shows loupe view of hematoxylin and eosin staining of the specimen. Furthermore, hematoxylin and eosin staining (**C**–**E**) revealed that most of the lesion consisted of conventional squamous cell carcinoma (SCC) forming solid nests with obvious keratinization (**C**). There were typical spindle cells containing pleomorphic nuclei observed adjacent to the conventional SCC foci (**D**) as well as atypical spindle cells that often incorporated neutrophils within their cytoplasmic vacuoles (**E**). Immunoperoxidase staining showed neutrophil elastase expression (**F**), as well as intense LAMP-1 (**G**) and cathepsin B (**H**) mainly localized around the vacuoles that contained the neutrophils. Panels **E**–**H** show serial sections. Scale bars: 10 mm (**B**), 500 μm (**C**), 200 μm (**D**), 50 μm (**E**–**H**), and 10 μm (inset in **E**–**H**)

The hemimandibulectomy specimen had a protruding tumor that exhibited an ulcerated surface and that measured 23 × 15 mm from the gingiva to buccal mucosa (Fig. 5A). The location of this tumor was not the same site of the previous tumor. On the cut surface, there was a whitish and solid lesion invading the mandibular bone (Fig. 5B, left). Histologically, most of the lesion was composed of conventional SCC, measured 45 × 25 × 15 mm in size, and invaded the mandibular bone extensively (Fig. 5B, right and Fig. 5C). The spindle cells were distributed near the SCC foci, mostly in the remaining polypoid segment (Fig. 5D). Because the atypical spindle cells were concomitant with the conventional SCC component, we finally diagnosed the lesion as SCSCC. The tumorous spindle cells frequently contained neutrophils within the cytoplasmic vacuoles (Fig. 5E, F), forming cell-in-cell figures. Such figures were much rarer in the conventional SCC cells. Intense staining for lysosomal-associated membrane protein 1 (LAMP-1) and cathepsin B, which are both lysosomal markers, were observed around the vacuoles containing the neutrophils (Fig. 5G, H). This suggested that such cell-in-cell figures may result from the phagocytosis of neutrophils rather than from emperipolesis. The patient has had no evidence of recurrence 5 years after the surgery.

## Discussion and conclusions

SCSCC is an uncommon variant of SCC comprising carcinoma cells that exhibit sarcomatoid differentiation following epithelial–mesenchymal transition (EMT) [1, 13]. Our patient had gingival SCSCC exhibiting prominent phagocytic figures containing neutrophils, although it was difficult to reach a definitive diagnosis based on the biopsy specimen. Moreover, we postulated that the neutrophils within the tumor cells were phagocytized by tumor cells and were not a result of emperipolesis.

The diagnosis of SCSCC is sometimes challenging when squamous components such as conventional SCC, carcinoma *in situ*, and epithelial dysplasia are absent from the specimens, particularly in those that are small. In our patient, the hemimandibulectomy specimen exhibited typical SCSSC features, which grossly showed typical polypoid protrusions and a biphasic histology composed of a spindle cell component admixed with a conventional SCC component. Meanwhile, the biopsy specimen contained only a spindle cell component covered by squamous epithelia without obvious atypia. Therefore, we sought to differentiate between SCSCC and neoplastic or reactive mesenchymal lesions when examining the biopsy specimen. High-grade sarcoma, including undifferentiated pleomorphic sarcoma, was strongly suspected as a mesenchymal neoplasm, given no obvious evidence of osteogenic, muscular, or adipogenic differentiation. The negative S-100 immunoreactivity indicated that a malignant melanoma was less likely, because S-100 positivity is observed in more than 90% of those, including amelanotic melanoma [14]. Additionally, radiation-induced stromal atypia [15] was also considered as a differential diagnosis.

Combined immunohistochemistry for epithelial markers is helpful for distinguishing SCSCC from other lesions [15], as exemplified by our patient. Most markers of epithelial differentiation, including pan-cytokeratin (clone AE1/AE3), were absent from our patient’s lesion; only p63 was detected in the spindle cell nuclei. Thompson *et al*. found that spindle cell component immunoreactivity for at least one epithelial marker was observed in 68% of their patients with laryngeal SCSCC [16]; this was consistent with the findings of Viswanathan *et al.*, who showed that 53% of SCSCCs of the head and neck region were positive for at least one epithelial marker [3]. Furthermore, p63 is reported to be a particularly useful epithelial marker for differentiating head and neck SCSCC from other mesenchymal lesions, given that this protein was found to be expressed in 63% of SCSCCs but in only 9% of various sarcomas and 5% of benign spindle cell lesions [17]. Additionally, reactive spindle cell lesions could be ruled out because of the strong expression of both p53 and Ki-67 in our patient’s tumor.

In the head and neck region, the oral cavity is a common site of occurrence for SCSCC [3, 4]. Specifically, the lower lip, tongue, and alveolar ridge are the commonly affected subsites [18]. Therefore, pathologists should consider SCSCC as a differential diagnosis in the presence of spindle cell lesions. Ellis and Corio reported that a history of therapeutic irradiation of the site of a subsequent SCSCC might be one of the etiologies [18]; this was the case for our patient: he received radiation against SCC of the buccal mucosa 15 years before. SCSCC is an aggressive malignancy and tends to recur locally; however, the prognosis is similar to that of conventional SCC [4]. When SCSCC occurred as a second primary tumor in a different part of the oral cavity, it has more favorable clinical outcomes than those occurring as a recurrent tumor in the same site of the primary tumor [19].

The phagocytosis of neutrophils by tumor cells is not a well-understood phenomenon; its biological significance and underlying mechanisms remain unclear. Malignant tumor cells have been shown to possess phagocytic activity against neighboring tumor cells [5, 12] as well as other types of cells, including neutrophils [7–9]. To date, phagocytized neutrophils have been observed in gastric adenocarcinoma [7, 20], salivary duct carcinoma of the parotid gland [21], and even in cytological specimens from diverse malignant tumors, including laryngeal SCC [22]. However, to the best of our knowledge, there have been no reports of neutrophil phagocytosis in SCSCC. Caruso *et al.* visualized ultrastructural cellular changes using electron microscopy and showed evidence that apoptotic neutrophils are actually incorporated into the cytoplasm of gastric adenocarcinoma cells, suggesting that tumor cells ingest such apoptotic bodies [20]. Our finding of intense staining of LAMP-1 (a lysosomal membrane protein) and cathepsin B (a lysosomal proteolytic enzyme) localized around neutrophils within tumor cells is also evidence of the active phagocytic and digestive abilities of malignant tumor cells. Spindle cell components are considered a result of EMT [13], which is induced by the Rac1 pathway [23]. Therefore, Rac1 activation may be an underlying mechanism of neutrophil phagocytosis, as well as of apoptotic cell phagocytosis by tumor cells [12]. In addition, the frequency of phagocytosis by tumor cells is higher in moderately differentiated oral SCC than well-differentiated ones [24], and unlike primary melanoma cells, metastatic melanoma cells have a phagocytic ability [8]. Thus, the presence of neutrophils phagocytized by tumor cells appears to be a potent indicator of malignancy. Further investigation is necessary to determine the molecular mechanisms that underlie neutrophil phagocytosis.

In conclusion, we reported a rare case of SCSCC exhibiting prominent phagocytosis of neutrophils by tumor cells. Combined immunohistochemistry for epithelial markers, such as p63, is useful in differentiating SCSCC from other spindle cell lesions, and the phagocytic figures containing neutrophils might support a diagnosis of malignancy.

## Data Availability

The data are available from the corresponding author upon reasonable request.
